# Factors Associated with Older Peoples′ Perceptions of Dignity and Well-being at Residential Care Facilities.

**DOI:** 10.1093/geroni/igab046.3488

**Published:** 2021-12-17

**Authors:** Charlotte Roos, Moudud Alam, Anna Swall, Anne-Marie Boström, Lena Hammar

**Affiliations:** 1 Dalarna University, Falun, Dalarnas Lan, Sweden; 2 Dalarna University, Borlänge, Dalarnas Lan, Sweden; 3 Karolinska Institutet, Karolinska Institute, Stockholms Lan, Sweden; 4 Mälardalen University / Dalarna University, Västerås, Vastmanlands Lan, Sweden

## Abstract

Dignity and well-being should be promoted in care of older people living at residential care facilities (RCFs). In addition, care should be person-centred. Dignity and well-being can be interpreted as person-centred outcomes. Older people living at RCFs experience a lack of dignity and well-being. To promote this, it is important to understand the associated factors to target. The aim of this study was to examine the associations between perceived dignity and well-being and factors related to attitudes of staff, the care environment and individual issues (age, gender, self-rated health and dementia) among older people living at RCFs. A national cross-sectional study was conducted retrospectively. All older people 65 years and older (n=71,696) living at RCFs in 2018 were invited to respond to the survey. The survey included the areas: self-rated health, indoor-outdoor-mealtime environment, performance of care, treatment from staff, safety, social activities, availability of staff and care in its entirety. Age, gender and diagnosed dementia were collected from two national databases. Data was analysed using ordinal logistic regression models. The result indicated that respondents who had experienced disrespectful treatment, who did not thrive in the indoor-outdoor-mealtime environment, who rated their health as poor and respondents with dementia had higher odds of being dissatisfied with dignity and well-being. There is a need to improve the prerequisites of staff regarding respectful attitudes and to improve the care environment. The Person-centred Practice framework, targeting the prerequisites of staff and the care environment, can be used as a theoretical framework for designing future improvements.

